# VR for Mental Health Interventions at Home: Feasibility and Guiding Principles for Success

**DOI:** 10.1192/bjo.2025.10241

**Published:** 2025-06-20

**Authors:** Bhavana Thummala

**Affiliations:** Imperial College London, London, United Kingdom

## Abstract

**Aims:** Virtual reality (VR) is becoming readily recognised as a transformative force in healthcare. In recent times there have been rising waiting lists, barriers to accessing care and a need to rapidly scale treatment provision for mental health conditions including depression, anxiety, anorexia, and phobias. As a result, the feasibility and design of current home-based VR mental health treatments has been evaluated to synthesise cohesive guiding principles for further development and implementation of Virtual Reality technologies.

**Methods:** A multi-method approach was employed, utilising both secondary data from literature reviews and primary data collection through semi-structured expert interviews and surveys to gain insights from all potential stakeholders. These were thematically coded through the COM-b behavioural model to produce a qualitative analysis uncovering key themes hindering and facilitating the adoption of VR technology. Survey data was subject to quantitative analysis to understand attitudes and guide our recommendations.

**Results:** Analysis of the results ascertained the key barriers and opportunities to provide improved VR mental health services for patients. The two main overarching themes found were: optimisation of the design of technology, and provision of a supportive deployment environment. Within design a significant theme was immersion; This allows simulated environments to replicate the benefits of in-vivo interventions. Presence, a subtheme, is key to supporting immersion and can be thought of as the suspension of disbelief in one’s surroundings, allowing for the mimicking of the effects of in vivo exposure. Personalisation and interaction also contributed to enhancing the design. For the higher theme of creating a supportive deployment environment, the main considerations were acknowledging inequalities regarding both technical barriers and financial challenges.

A patient centered framework was developed to support engagement and adoption of VR, using the COM-B model. The model shows the interaction between the capability to participate in an action, the opportunity, which is determined by external factors that allow behavioural change, and the motivation to implement these changes.

**Conclusion:** The evidence for VR’s therapeutic efficacy on home mental health is growing rapidly, however, translating evidence-based practices into the NHS can be a lengthy process. A theory driven framework of practical recommendations is vital for implementing VR interventions. Focus should be centred on holistic design and prioritization of facilitators, which address barriers under multiple domains to support patient and clinician confidence, and thus the successful adoption of VR.

