# VR for Mental Health Interventions at Home: Feasibility and Guiding Principles for success – CORRIGENDUM

**DOI:** 10.1192/bjo.2025.10886

**Published:** 2025-10-10

**Authors:** William Benjamin Clare, Shamita Suresh, Bhavana Thummala, Nimath Sumaithaa Shadhuly, Kashir Ahmed Raza, Bhone Hein Kyaw

In the original publication of this abstract, multiple names were missing from the author list. The co-authors of this abstract are:

William Benjamin Clare, Shamita Suresh, Bhavana Thummala, Nimath Sumaithaa Shadhuly, Kashir Ahmed Raza, and Bhone Hein Kyaw

The abstract has been updated to include these authors, and this corrigendum published.
